# Molecular Mechanisms of Pathogenic Fungal Virulence Regulation by Cell Membrane Phospholipids

**DOI:** 10.3390/jof11040256

**Published:** 2025-03-26

**Authors:** Yitong Li, Hongchen Wang, Hengxiu Wang, Tianming Wang, Daqiang Wu, Wenfan Wei

**Affiliations:** 1 Department of Pathogenic Biology and Immunology, College of Integrated Chinese and Western Medicine (College of Life Science), Anhui University of Chinese Medicine, Hefei 230038, China; l18131414122@163.com (Y.L.); h.c.wang@ahtcm.edu.cn (H.W.); whx314413@163.com (H.W.); wtm1818@163.com (T.W.); 2 Institute of Integrated Traditional Chinese and Western Medicine, Anhui University of Chinese Medicine, Hefei 230038, China

**Keywords:** pathogenic fungi, virulence, cell membrane, phospholipid composition, molecular mechanisms, antifungal therapy

## Abstract

Pathogenic fungi represent a growing concern for human health, necessitating a deeper understanding of their molecular mechanisms of virulence to formulate effective antifungal strategies. Recent research has increasingly highlighted the role of phospholipid components in fungal cell membranes, which are not only vital for maintaining cellular integrity but also significantly influence fungal pathogenicity. This review focuses on the impact of membrane phospholipid composition on fungal growth, morphogenesis, stress responses, and interactions with host cells. To be specific, membrane phospholipid composition critically influences fungal virulence by modulating growth dynamics and morphogenesis, such as the transition from yeast to hyphal forms, which enhances tissue invasion. Additionally, phospholipids mediate stress adaptation, enabling fungi to withstand host-derived oxidative and osmotic stresses, crucial for survival within hostile host environments. Phospholipid asymmetry also impacts interactions with host cells, including adhesion, phagocytosis evasion, and the secretion of virulence factors like hydrolytic enzymes. These adaptations collectively enhance fungal pathogenicity by promoting colonization, immune evasion, and damage to host tissues, directly linking membrane architecture to infection outcomes. By elucidating the molecular mechanisms involved, we aim to underscore the potential of targeting phospholipid metabolic pathways as a promising avenue for antifungal therapy. A comprehensive understanding of how membrane phospholipid composition regulates the virulence of pathogenic fungi can provide valuable insights for developing novel antifungal strategies.

## 1. Introduction

The structure of the cell membrane is fundamental to the physiology of fungi, serving as a barrier that regulates the entry and exit of substances, and facilitating communication with the external environment [1]. The fungal cell membrane is primarily composed of a lipid bilayer, which includes various phospholipids, sterols, and proteins that contribute to its integrity and functionality. Among these, ergosterol is a key component that is similar to cholesterol in mammalian cells, providing membrane fluidity and stability [2,3]. This unique composition not only supports essential cellular processes but also influences the virulence of pathogenic fungi [2,4,5]. *erg11* encodes a key enzyme for ergosterol synthesis in *Candida albicans*, and changes in ergosterol levels in *erg11∆/∆* mutant strains significantly affect *C. albicans* virulence, including cell surface hydrophobicity, biofilm-forming capacity, and hyphae-forming ability [3]. The diversity of phospholipid components in fungal membranes is notable, with variations in fatty acid saturation and head group composition that can affect membrane properties and interactions with antifungal agents [6]. Understanding these structural features is crucial, as they are intimately linked to the pathogenicity of fungi and their ability to cause disease in humans. In particular, the modulation of lipid composition can alter the susceptibility of fungi to host immune responses and antifungal treatments, highlighting the importance of membrane lipids in fungal virulence [7].

The pathogenicity of fungi is often associated with their ability to adapt to various environmental stresses and evade host defenses. For instance, species such as *C. albicans* and *Aspergillus fumigatus* exhibit complex virulence traits, including the ability to form biofilm [8], secrete hydrolytic enzymes [9], and produce secondary metabolites that can suppress host immune responses [10]. These virulence factors are influenced by the composition and organization of the fungal cell membrane, which can modulate interactions with host cells [7] and the effectiveness of antifungal therapies. The intricate relationship between lipid composition, membrane dynamics, and fungal virulence underscores the need for further research into the molecular mechanisms by which phospholipid components regulate pathogenicity.

Recent studies have begun to elucidate the molecular mechanisms by which phospholipid components modulate fungal virulence. For example, Chen et al. demonstrated that phosphatidylserine synthase and decarboxylase are critical for maintaining cell wall integrity and virulence in *Candida albicans*, linking phospholipid metabolism to pathogenicity, and Konarzewska et al. (2019) revealed that phosphatidylserine synthesis is indispensable for *Cryptococcus neoformans* survival, highlighting its potential as a therapeutic target in fungal infections [11,12]. Moreover, alterations in the composition of membrane phospholipids can influence the activity of membrane-bound proteins involved in signaling pathways that regulate virulence traits [13]. Additionally, the role of lipid transporters in maintaining membrane integrity [14,15] and facilitating the adaptation of fungi to environmental stresses [14,16] has gained attention as a potential target for novel antifungal strategies. By understanding how phospholipid components impact fungal physiology and pathogenicity, researchers can develop innovative approaches to combat fungal infections and improve therapeutic outcomes.

## 2. Classification and Function of Phospholipid Components

**Figure 1:**
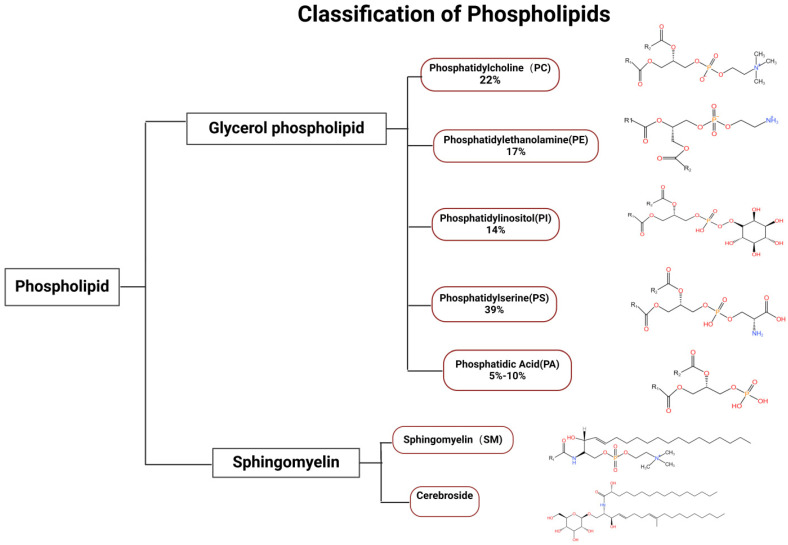
Classification of Phospholipids. Phospholipids include two major categories, glycerophospholipids and sphingolipids, and glycerophospholipids are divided into phosphatidylcholine (PC), phosphatidylethanolamine (PE), phosphatidylinositol (PI), phosphatidylserine (PS) and its phosphorylated derivatives, and phosphatidic acid (PA). In *Candida albicans* [24], PS is the most abundant, accounting for about 39%, with the remaining PE, PI, PC, and PA accounting for 17%, 14 %, 22%, and 5–10%, respectively. Sphingolipids in fungi are minor membrane phospholipids and the distribution of the content has not been clarified. The types and contents of phospholipids varied significantly among different fungi.

Phospholipids are essential components of all biological membranes, playing a critical role in maintaining membrane integrity and fluidity. They can be classified into various types based on their structure and function, including glycerophospholipids, sphingolipids and other phospholipids, for example, phosphatidic acid (PA) and diacylglycerol (DAG) (Figure 1) [15]. There is also a phospholipid with multiple head groups which is cardiolipin. It consists of two phosphatidyl groups (each with a phosphate head) linked by a central glycerol, forming a dimeric structure with four fatty acyl chains and two distinct phosphate-containing polar regions. This unique architecture allows cardiolipin to play critical roles in mitochondrial membrane integrity, energy metabolism, and membrane protein stabilization. It is predominantly found in bacterial and mitochondrial inner membranes. Glycerophospholipids, the most abundant class, consist of a glycerol backbone, two fatty acid tails, and a phosphate group that may be modified by different head groups, such as choline, ethanolamine, or serine. The primary types of phospholipids include phosphatidylcholine (PC), phosphatidylethanolamine (PE), phosphatidylserine (PS), and phosphatidylglycerol (PG), each differing in their head groups and fatty acid chains. This structural diversity allows phospholipids to perform various biological functions, including forming lipid bilayers, serving as signaling molecules [17], and participating in cellular processes such as apoptosis [18,19] and membrane trafficking [20]. For instance, phosphatidylserine (PS) is known for its role in apoptosis, where its externalization on the cell surface signals macrophages to engulf dying cells, thus maintaining tissue homeostasis [20,21]. In fungal membranes, sphingolipids form microdomains with sterols, called lipid rafts, which are essential for growth, cell polarity establishment, mycelium formation, and ultimately virulence. In *Aspergillus lcbA*, encoded serine palmitoyltransferase (SPT) catalyzes the generation of 3-ketodihydrosphingolipids from serine and palmitoyl coenzyme [22]. Such sphingolipids are capable of influencing polarized growth and mycelial formation in *Aspergillus*. Cell polarity is an important marker of fungal virulence [23], so SPT may influence virulence. The pathogenicity of fungi is usually associated with their impact on various biological functions and their involvement in cellular processes, and there is a need to further investigate the molecular mechanisms by which phospholipid components regulate pathogenicity.

### 2.1. Biosynthetic Pathways of Glycerophospholipids in Fungal Cells

**Figure 2:**
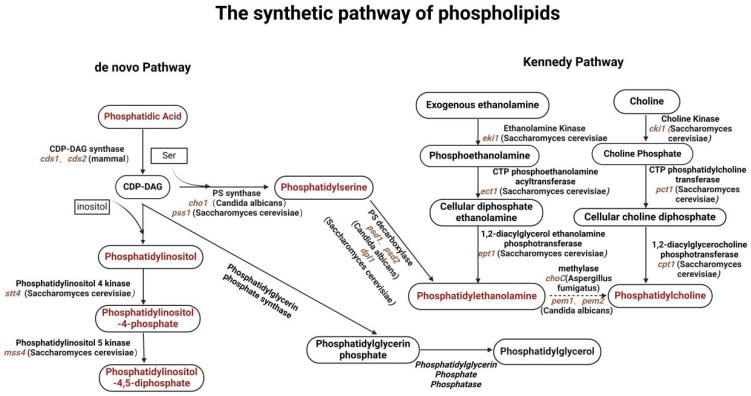
The synthetic pathway of phospholipids. Fungi synthesize phospholipids via the endogenous pathway and the Kennedy pathway. The most common phospholipid precursors are phosphatidic acid (PA) and CDP-DAG, followed by enzymatic conversion of CDP-DAG to phosphatidylinositol (PI), phosphatidylserine (PS), and phosphatidylglycerol (PG) in the endogenous pathway. Endogenously generated PS can also be further decarboxylated to produce phosphatidylethanolamine (PE), which is further followed by phosphatidylcholine (PC) in the presence of methylases. PE and PC can also be introduced into the cell and converted to PE and PC via the Kennedy pathway by exogenous ethanolamine (Etn) and choline (Cho). Red markings represent phospholipids, italics are genes encoding key enzymes, and parentheses indicate the species within which the gene is present.

The biosynthesis of glycerophospholipids in fungi involves several key pathways that integrate various metabolic precursors. The Kennedy pathway [25] is the primary route for synthesizing phosphatidylcholine and phosphatidylethanolamine, where diacylglycerol (DAG) [26] is formed from glycerol-3-phosphate and fatty acetyl-CoA (Figure 2). Additionally, fungi can utilize alternative pathways, including the remodeling of existing phospholipids, which can be crucial for adapting to environmental changes or stress conditions. Recent studies have shown that certain signaling pathways, such as the Cell Wall Integrity (CWI) pathway [13], also play a role in regulating phospholipid biosynthesis, linking cell wall integrity to membrane composition and function [27], and that the integrity of the cell membrane is critical for both the invasive ability and virulence of fungi, while the mechanism of action of many antifungal drugs is also linked to phospholipid biosynthesis. For example, azoles can interfere with ergosterol synthesis by inhibiting the lanosterol-14α-demethylase, further affecting cell membrane integrity and function [28]. Phospholipids also play a central role in the morphological transformation of fungi, and in *C. albicans* phospholipid biosynthesis can influence the transition from the yeast state to the mycelial state, a dimorphic transition that is closely related to the virulence of *C. albicans* [29]. Understanding the biosynthetic pathway of phospholipids is essential for the development of new antifungal drugs against fungal virulence.

### 2.2. Intracellular Transport Mechanisms of Glycerophospholipids

The intracellular transport of glycerophospholipids is a vital process that ensures the proper distribution and function of these lipids within the cell. Various mechanisms, including vesicular transport and non-vesicular transport, facilitate the movement of phospholipids between organelles [30,31,32]. For instance, lipid transfer proteins (LTPs) play an essential role in non-vesicular transport, transferring lipids directly between membranes without the need for vesicle formation [30]. Additionally, endosomes and Golgi networks are involved in the trafficking of phospholipids to their respective destinations, such as the plasma membrane or lysosomes [33,34]. Disruption of these transport mechanisms can lead to cellular dysfunction and cause reduced virulence and increased drug sensitivity, e.g., in *C. albicans*, the ABC transporter protein Mlt1p transports PCs into the vacuolar lumen thereby affecting lipid homeostasis and ultimately leading to reduced virulence [35], and in filamentous fungi, DnfAp, a member of the P4 ATPase family, is involved in spore polarization and growth [15].

### 2.3. Cellular Functions of Phospholipids

Phospholipids are not merely structural components of membranes; they are actively involved in various cellular processes. Their amphipathic nature allows them to form lipid bilayers, which serve as barriers to protect cellular contents while facilitating selective permeability. Furthermore, phospholipids are key players in cell signaling pathways, acting as precursors for bioactive molecules such as prostaglandins and phosphoinositides, which regulate diverse physiological responses [36,37]. For example, phosphatidylinositol 4,5-bisphosphate (PIP2) is crucial for the activation of various signaling proteins and ion channels, influencing processes such as muscle contraction and neurotransmitter release [38,39,40]. Additionally, phospholipids are involved in membrane fusion events [41], apoptosis, and autophagy [42,43], highlighting their versatility and importance in maintaining cellular homeostasis and responding to environmental changes. Understanding the multifaceted roles of phospholipids is essential for unraveling their contributions to cellular function and disease mechanisms (Table 1).

## 3. The Role of Membrane Phospholipid Composition in Fungal Virulence

In the composition of cell membranes, phospholipids are the primary lipid constituents, including phosphatidylcholine (PC), phosphatidylethanolamine (PE), phosphatidylinositol (PI), and phosphatidylserine (PS) as well as phosphatidic acid (PA). These not only serve as structural components of the cell membrane but also participate in signaling pathways that influence fungal growth [56,57], morphological development [58], stress responses [59,60], and interactions with the host environment [61,62] (Table 1). Changes in the phospholipids’ homeostasis have been shown to affect the pathogenicity of fungi, highlighting the importance of understanding the molecular mechanisms involved in phospholipid metabolism and regulation [10,63]. The formation of lipid bilayers leads to an uneven distribution of charge on the cytoplasmic membrane. The negatively charged phosphoinositide family (PIPs), phosphatidic acid (PA), and phosphatidylserine (PS) contribute to the asymmetric distribution, resulting in an unequal distribution of negative charges on the cytoplasmic membrane [64]. This uneven distribution makes it easier for positively charged ions or proteins to interact with phospholipids [64]. Studies in fungal cells have shown that the synthesis and distribution of these negatively charged phospholipid components on the inner side of the cytoplasmic membrane can also impact the polarized growth of the cells [45]. Some pathogenic fungi, such as *C. albicans* and *A. fumigatus*, have also been shown to show an influence of their pathogenicity by the polarity of cell growth [65,66] (Table 1). Therefore, the membrane phospholipid composition plays a crucial role in fungal virulence.

### 3.1. Phosphoinositides (PIPs)

The phosphatidylinositol family (PIPs) is obtained by phosphorylation of phosphatidylinositol (PI), and in *C. albicans*, the phosphatidylinositol-3-kinase, Vps34p, has lipid kinase activity that converts PI to phosphatidylinositol [67]. To determine whether inositol phosphate is associated with virulence, Juliane Günther et al. [67] tested the lipid kinase-deficient strain CAV9 in a mouse model of systemic Candida infection. It was found that all mice infected with the mutant strain survived for three weeks, while wild-type mice infected with the same number of cells died two days later, demonstrating the important role of inositol phosphate in virulence. Vps34p was also able to influence the secretion of aspartic protease, whose activity is closely related to virulence [68]. In vivo, aspartic protease promotes the adhesion of *C. albicans* to epithelial cells. In contrast, in a mouse model of systemic infection, the *vps34* mutant showed reduced adhesion to mouse L929 fibroblasts, resulting in no virulence of the mutant [69]. Morphological transformation is one of the important virulence factors of *C. albicans*, and PI(4)P in the Golgi and PI(4,5)P_2_ in the plasma membrane are required for the morphological transformation of *C. albicans* from the yeast to the mycelial state. Phosphatidylinositol-4-phosphate in the Golgi [70] is involved in the transformation of *C. albicans* from the yeast to the mycelial state, but at the plasma membrane, stt4 encodes a PI-4 kinase, and despite reduced levels of phosphatidylinositol-4-phosphate, the mutants were able to generate budding tubes and transform to the mycelial state. Previous studies have shown that the PI(4)P-5-kinase activity of *C. albicans* reaches its peak during the transition period of morphological transformation [71], indicating that PI(4,5)P_2_ may be involved in this transition period. At the same time, the content of PI(4)P and PI(4,5)P_2_ on the cell membrane of mutant strains decreased, which not only affected the polar growth of hyphae but also led to invasive growth defects [54]. This indicates that PI(4,5)P_2_ is necessary for the morphological transformation of *C. albicans*, and a steep concentration gradient of PI(4,5)P_2_ is crucial for filamentous growth.

Among the signaling pathways responsible for regulating fungal filamentous growth, mitogen-activated protein kinase (MAPK) is one of the important pathways in eukaryotic signaling, which can participate in regulating the differentiation of filamentous cells and the development of biofilms. Aurélia Vernay et al. [54] found that phosphatidylinositol phosphate can play a signaling role in the MAPK pathway, thereby participating in the regulation of yeast’s morphological changes. For example, the kinase Pik1p regulates the HOG pathway and pheromone response at the Ste11p level [72]; the activation of the yeast mating pathway also requires recognition of PI(4,5)P_2_ in order to recruit the mating pathway-specific scaffold Ste5p to the PM [73]. In *C. albicans*, the homologous differentiation MAPK pathway (Cek1p) regulates hyphal growth and biofilm formation [74].

The key lipid phosphatidylinositol (PI) phosphorylates the inositol ring at different positions through the lipid kinase family, giving organelles a specified PIP combination. In *Saccharomyces cerevisiae*, two types of kinases are expressed to produce PI(4)P: PiK1p is produced on the Golgi apparatus, and Stt4P is produced on the PM. The phosphorylation level of Kss1p in the MAPK pathway reflects the growth status of hyphae. Hema Adhikari et al. [55] conducted temperature-sensitive tests on PI kinases Pik1-83, Stt4-4, and Mss4-102 in the SEY6210 strain, and the conditional mutants Pik1-83, Stt4-4, and Mss4-102 were tested for temperature sensitivity. Pik-83, Stt4-4, and Mss4-102 showed reduced levels of Kss1p phosphorylation at 37 °C, indicating that PI(4)P production is necessary for activation of the filamentous growth MAPK pathway. The membrane-related regulatory factors of the MAPK pathway in filamentous growth include Msb2p, Sho1p, and Cdc42p [61,75], and PI(4)P is involved in localizing the membrane-related regulatory factors of the MAPK pathway. Thus, reduced MAPK activity in PI(4)P kinase mutants suggests that PI(4)P kinase is a key regulator in membrane transport [76]. Before transporting substances to the PM site, PI needs to be modified to PI(4)P on the Golgi apparatus [77,78]. The signal defects in Pik1-83 and other PI(4)P mutants are most likely due to incorrect localization of the PM protein in the MAPK pathway. The transmembrane protein encoded by Sho1p serves as an adapter for the MAPK pathway [79,80] and HOG pathway [81] in filamentous growth, and it also exhibits misplacement in Pik1-83, Stt4-4, and Mss4-102 mutants [50]. Cdc42p is a rho-type GTPase that regulates the MAPK pathway involved in filamentous growth, among other functions [82,83]. This protein is typically located on the plasma membrane. Cdc43p GFP also exhibits misplacement in *pik1-83* mutants, *stt4-4* mutants, and *mss4-102* mutants compared to the wild type. Previous studies have suggested that the localization defects of MAPK regulatory proteins in filamentous growth may be due to decreased protein stability [84]. However, Hema Adhikari et al. [55] confirmed that there is a defect in the PI(4)P-dependent transport pathway components to PM in PI kinase mutants, not due to loss of protein abundance, but rather related to the activation of the MAPK pathway in filamentous growth. Thus, PI(4)P production is necessary for activation of the filamentous growth MAPK pathway, possibly by affecting the localization of membrane-associated regulators. Studies on filamentous growth in S. cerevisiae can provide information on the genetic basis of fungal behavior that is equally applicable in pathogens such as *C. albicans*. In *C. albicans*, PI signaling is involved in the regulation of filamentous growth [54,71], which can be explained at least at the level of the MAPK pathway.

In addition, inositol phosphate is also an important membrane component closely related to PKC activity. *stt4* and *mss4* mutants with PKC-MAPK signaling defects lead to reduced inositol synthesis [85], resulting in a reduced recruitment of Rho1-GTPase GEF Rom2 at polarized growth sites, ultimately exhibiting reduced CWI signaling because PIPs interact with the pH domain of Rom2 and ensure its correct membrane localization [86,87]. The CWI sensor Rom2 can increase the concentration of Rho1 bound to active GTP, thereby activating PKC1, which participates in regulating the cAMP-PKA signaling cascade and ultimately affects the morphological transformation of *C. albicans* [88].

### 3.2. Phosphatidic Acid (PA)

**Figure 3:**
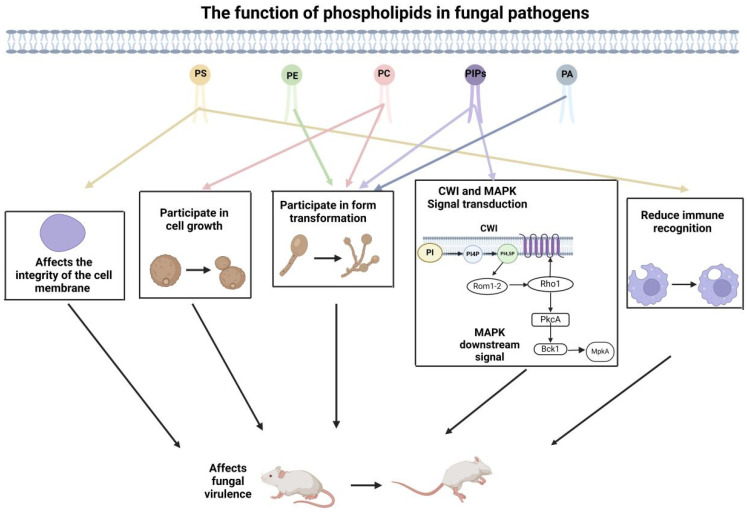
The function of phospholipids. This figure mainly lists the functions of phospholipids involved in influencing virulence, such as participating in cell growth, morphological transformation, and signal transduction. PS—Phosphatidylserine, PE—Phosphatidylethanolamine, PC—Phosphatidylcholine, PIPs—Phosphoinositides, PA—Phosphatidic Acid.

Phosphatidic acid (PA) is the core component of lipid metabolism and the premise of synthesizing other glycerophosphates. The role of PA in yeasts and filamentous fungi is mainly studied by studying the gene PLD1 for PA synthesis. In *C. albicans*, PLD1 is a gene encoding phospholipase D, which not only affects its morphological transformation but also is related to virulence [89] (Table 1; Figure 3). In the Candida mouse model, it was proved that the deletion of PLD1 reduced the toxicity. Experiments showed that the PLD1 mutant retained the ability to form mycelium and reduced the toxicity [29]. In *A. fumigatus*, PLD has three subtypes, PLD, PLD1, and PLDA, one of which regulates the internalization of *A. fumigatus* spores in lung epithelial cells and is related to the virulence of *A. fumigatus* [10]. Li et al. [10] infected A549 lung epithelial cells with wild-type strains and PLD mutant strains. The data showed that the presence of PA significantly promoted the strain to internalize into lung epithelial cells in a dose-dependent manner (Table 1). PA is an important signaling molecule that may promote the internalization of *A. fumigatus* strains by regulating signaling pathways within host cells, such as the PLD signaling pathway. Phospholipases are composed of various enzymes, including phospholipase A (PLA), phospholipase B (PLB), phospholipase C (PLC), and phospholipase D (PLD), which are further classified into different subtypes [52]. In *C. albicans,* phospholipase D1 (PLD1) hydrolyzes membrane phospholipids (PC, PE, PI, and PC) to phosphatidic acid (PA) and basic head group, and PA is then hydrolyzed by PLA1 to DAG [62], participating in the morphological transformation from yeast to mycelial growth [89]. Both PA and DAG are signal molecules that can regulate membrane structure and function because they can act as lipid signaling molecules by altering membrane curvature, fluidity, and domain organization, thereby shaping membrane architecture for processes like vesicle trafficking or fusion [90]. They also directly recruit signaling proteins (e.g., Raf kinases for PA) or activate enzymes (e.g., PKC for DAG), linking structural membrane changes to functional outputs such as cell growth, secretion, or stress responses [13,91].

The process of yeast to mycelial morphological transformation is controlled by multiple parallel signaling pathways, which combine specific stimuli with several transcription factors. PLD1 plays a role in at least one signaling pathway of morphological transformation [29]. When yeast cells began to form embryonic tubes, PLD1 activity was stimulated, and exogenous phospholipase D stimulated this morphogenesis in the form of a partial peanut-purified enzyme [89]. Dolan et al. [29] cultured PLD1 homozygous mutants in solid spider medium and serum medium and found that they could not form hyphae on solid spider medium, but were able to form hyphae in the presence of serum, suggesting that certain specific stimuli (e.g., serum) were able to induce the involvement of PLD1 in morphological transformations, and this also confirms that PLD1 is involved in morphological transformations in the presence of certain stimuli. At the same time, *pld1* mutation can affect the virulence of the strain, and the mutant with *pld1* deletion showed significantly reduced virulence in two immunodeficient mice. No fatal infection was observed in bg/bg-nu/nu and transgenic mice inoculated with 10^6^ colonies of *pld1* mutant cells orally. However, all mice died within 42 days after inoculation with the same dose of wild-type yeast cells [92]. Therefore, the *pld1D* mutant showed significantly reduced virulence while retaining the ability to form mycelium in vivo.

### 3.3. Phosphatidylserine (PS) and Phosphatidylethanolamine (PE)

Phosphatidylserine (PS) has a glycerol backbone and two fatty acid chain tails like other phospholipids but differs in that the serine head group of PS is negatively charged and is predominantly located in the inner plasma membrane. Knocking out the gene encoding PS synthase will lead to abnormal cell polarity. *cho1* encodes PS synthase in *C. albicans*. In the *C. albicans* mouse infection model, the PS synthase *cho1* deletion mutant cannot cause infection, while mice infected with the wild-type or *cho1∆/∆::cho1* recombinant strain die within two weeks [11], indicating that PS is necessary for the virulence of *C. albicans* [46]. The absence of PS synthesis increases the exposure of β (1-3)—glucan within the cell wall, making it more easily recognized by host immune cells [7]. PS is a precursor for the production of phosphatidylethanolamine (PE), which in the endogenous pathway is catalyzed by PS decarboxylase (PSD) to produce PE. Mice were infected with embryo spores of 10^6^ *psd1∆/∆* or *psd2∆/∆* single mutants and *psd1∆/∆* and *psd2∆/∆* double mutants. The single mutant had virulence, and the performance was similar to that of the wild type, while the virulence of the double mutant was significantly reduced. This indicates that the toxicity may also be related to the loss of PE [11]. The *psd1∆/∆* and *psd2∆/∆* double mutants blocked PE synthesis but not PS synthesis. *cho1∆/∆* mutants were non-toxic, whereas they remained partially toxic in the *psd1∆/∆* and *psd2∆/∆* double mutants, suggesting that PS exhibits an additional role in toxicity, in addition to acting as a precursor to PE [11,46].

Chen et al. [11] found that *cho1∆/∆* mutants exhibit defects in cell wall integrity, mitochondrial function, and filamentous growth, and that *psd1∆/∆ psd2∆/∆* double mutants also have reduced levels of PE, similar phenotypes to *cho1∆/∆* mutants, but with greater virulence and fewer cell wall defects compared to *cho1∆/∆* mutants. Thus, the reduced virulence of *cho1∆/∆* and *psd1∆/∆*, *psd2∆/∆* mutants appears to be associated with their cell wall defects (Table 1).

*cdc50* is responsible for encoding the β-subunit of lipid turnover enzyme, which is involved in membrane phospholipid translocation, cell surface receptor signal transduction, vacuolar tissue, and maintaining the asymmetric distribution of phospholipids on the bilayer lipid membrane [93]. In *Staphylococcus*, Cdc50 affects the translocation of PS to maintain the asymmetry of bilayer membrane structure [94], while the localization of Cdc50 in *C. neoformans* is similar to that in other organisms [94,95]. Therefore, Cdc50 in *C. neoformans* may also affect the translocation of PS. PS on the cell surface is a signal for macrophages to recognize and internalize cells [96]. In the *cdc50∆* mutant strain, PS cell surface exposure increased and macrophage killing and virulence factors changed, so *cdc50∆* mutation may affect the virulence of *C. neoformans* [97]. To verify this conclusion, Wei [98] and others constructed a mouse model of aspiration cryptococcosis and infected it with wild-type, *cdc50∆* mutant, and supplementary strains. It was observed that mice infected with wild-type strains died after around 20 days, while mice infected with *cdc50∆* mutant strains remained healthy for 60 days after inoculation. At the same time, seven days after infection with the *cdc50∆* mutant, the cryptococcal cells in the lung were completely removed, while the fungal load in the brain did not change significantly. When HE staining or GMS staining was used in the lung sections of mutant infection, no cryptococcal cells were observed, but abundant cryptococcal cells were observed in the lung sections of wild-type infection. Thus, the Cdc50 phospholipid translocator is critical for *C. neoformans* virulence in vivo, and PS exposed at the cell membrane surface has multiple effects on *C. neoformans*-host interactions (Table 1).

### 3.4. Phosphatidylcholine (PC)

Phosphatidylcholine (PC) is one of the main phospholipid molecules in biofilm, accounting for about 50% of the phospholipids in eukaryotic cell membranes. There are few studies on the synthesis of PC by filamentous fungi. The common method of PC synthesis is the generation of PE catalyzed by methylase. In the human pathogenic fungus *A. fumigatus*, Choc encodes methylase. To study the role of choc in the pathogenesis of *A. fumigatus*, Pan et al. [48] constructed WT, mutant and supplementary strains, and observed the survival of mice by tail vein injection. Experiments showed that almost all mice survived until the fifth day, and most mice died on the fifth to tenth day. The virulence of the mutant was much lower than that of the wild type. Then, the fungal load in the liver, spleen, and kidney of mice was detected. It was found that the spores in the liver and kidney were removed within 3–10 days, and the spores gradually transferred to the kidney for colonization. At the same time, the renal tissues of infected mice were selected for pathological examination on the third day, the thirteenth day, and the twenty-third day. The results showed that obvious inflammatory cell infiltration could be observed in WT and supplementary strains, but not in mutant strains, indicating that the deletion of choc reduced the virulence of *A. fumigatus* spores. The synthesis of PC will also affect the normal synthesis of GPI anchor protein [48]. GPI anchor points are located in the cell wall. Abnormal synthesis of GPI will inevitably affect the integrity of the cell wall [99]. At the same time, abnormal synthesis of GPI will also lead to mycelial growth inhibition and asexual development [100]. For pathogenic fungi, cell wall integrity and hyphal morphology play an important role in their virulence, which not only affects the invasive ability of fungi but also protects them from the impact of the host defense system [101,102] (Table 1; Figure 3). Therefore, the lack of PC will have a negative impact on the growth and virulence of *A. fumigatus*.

In *C. albicans*, lipid transporters participate in the transport of PC to affect the transformation of morphology. The ABC superfamily is one of the largest protein superfamilies, including seven subfamilies: ABCA, ABCB, ABCC, ABCD, ABCE, ABCF, and ABCG. ABC superfamily transporters have many functions, such as absorption, excretion, signal transduction, and pathogenicity [103]. For example, Cdr1p and Cdr2p of *C. albicans* are homologues of *S. cerevisiae* ScPdr5, both of which are phospholipid transporters, maintaining membrane asymmetry and integrity [35]. Cryptococcal transporters CnItr1A and CnItr3C not only transport inositol, but also affect virulence [104]. The absence of abcB in *A. fumigatus* could also reduce the toxicity [105]. The vacuolar transporter cgctr2 of the plant pathogen Colletotrichum gloeosporioides is involved in copper transport, affecting its germination and pathogenicity [106]. This article focuses on the ABC transporter Mlt1p of *C. albicans* and introduces the mechanism of the transporter in fungal pathogenicity. The ABC transporter Mlt1p of *C. albicans* is in the vacuolar membrane and specifically transfers PC to the vacuolar cavity. It has physiological effects, such as delayed endocytosis, the offset and isolation of reactive oxygen species [13], mycelial development defects, and reduced toxicity [35]. Mlt1p is an ATP-dependent ABC transporter that can transport PC into the vacuole, which affects the lipid balance. The specific mechanism is that Mlt1p located in the vacuolar membrane uses the energy generated by ATP hydrolysis to transport PC analogue NBD-PC into the vacuolar cavity. In addition, the deletion of mlt1 also affected the filamentous growth of *C. albicans*. By studying the mycelial development ability of the *MLT1* mutant in the liquid mycelial induction medium (serum, spider, and RPMI1640), it was found that mycelial formation was only observed in a few cells of the mutant after 60 min, and mycelia could be observed at later time points (120, 180, and 240 min). Therefore, the deletion of mlt1 delayed the mycelial growth. Morphological transformation is an important virulence factor of *C. albicans* (Table 1; Figure 3), and mutants with defects in hyphal formation show reduced virulence [35].

## 4. Conclusions

In recent years, phospholipid components have been found to have an important impact on the virulence regulation of pathogenic fungi. These phospholipids are not only an important part of the cell membrane but also play a key role in cell signal transduction, membrane fluidity, and interaction with the host immune system. By analyzing the functions of different phospholipids, researchers gradually revealed their multiple roles in fungal pathogenesis. This discovery not only enriches our understanding of fungal biology but also provides new ideas for the treatment of fungal infection.

However, there are still some limitations in the research on the relationship between phospholipids and fungal virulence. The results of different studies are often inconsistent, which may be related to the experimental conditions, fungal species, and their specific physiological state. Therefore, it is urgent for the scientific community to systematically compare and integrate these research results to have a more comprehensive understanding of the specific role and mechanism of phospholipids in fungal virulence. In addition, how to balance the views and findings of different studies will be an important task for future research.

It is also very promising to apply our understanding of the functions of membrane phospholipids to the diagnostic tests. For example, in mammalian cells, phosphatidylcholine (PC) depletion in colonic mucus is linked to ulcerative colitis pathogenesis, demonstrating that lipid profiling can reflect disease states [107]. Similarly, fungal-specific phospholipids (e.g., cardiolipin in mitochondrial membranes) or lipid-modifying enzymes (e.g., Tafazzin in Barth syndrome) might serve as biomarkers. Advanced techniques like mass spectrometry, used to characterize *Pseudomonas putida* phospholipids, could identify fungal lipid signatures [108]. Additionally, molecular dynamics modeling reveals how lipid membrane organization influences pathogenicity, providing a framework to study fungal membrane-targeted therapies. However, challenges persist, including distinguishing pathogen-derived lipids from host lipids and standardizing detection methods. Further research is needed to validate these mechanisms and develop lipid-based diagnostics for mycoses.

Future research should focus on several key issues. Firstly, the specific functions and mechanisms of different types of phospholipids in various pathogenic fungi should be discussed in depth. Secondly, the details of the interaction between phospholipids and the host immune system should be studied to reveal their potential regulatory role in the process of fungal infection. Finally, an exploration of intervention strategies based on phospholipids is needed to find new antifungal drugs or immunomodulators and improve clinical therapeutic effects.

## Figures and Tables

**Table 1:** The functions of phospholipids and phospholipids in toxicity.

Phospholipid	The Function of Phospholipids		Fungus
Phosphatidylserine (PS)	Membrane transport		*Candida albicans* [44]
	Regulating membrane fluidity		*Candida albicans* [44]
	Polarized growth		*Schizosaccharomyces pombe* [45]
	Affects fungal virulence	Reduce immune recognition	*Candida albicans* [7]
		Affect membrane integrity	*Cryptococcus neoformans* [46] *Candida albicans* [11]
		Decreased enzyme secretion ability	*Candida albicans* [46]
Phosphatidylethanolamine (PE)	Membrane transport		*Candida albicans* [6]
	Regulating membrane fluidity		*Candida albicans* [6,11]
	Affects fungal virulence	Cell wall integrity	
		Morphogenesis	*Candida albicans* [6] *Aspergillus fumigatus* [47]
Phosphatidylcholine (PC)	Participates in cell growth and metabolism		*Aspergillus fumigatus* [48]
	Maintain the integrity of cell membrane and cell wall		*Aspergillus fumigatus* [48]
	Regulating membrane fluidity		*Aspergillus fumigatus* [49]
	Affects fungal virulence	Morphogenesis	*Aspergillus fumigatus* [48] *Aspergillus oryzae* (non-fungal pathogen) [50]
Phosphatidic Acid (PA)	Key signaling molecules		*Saccharomyces cerevisiae* [51]
	Synthesis of secondary metabolites		*Ganoderma lucidum* [10]
	Affects fungal virulence	Regulating fungal internalization ability	*Aspergillus fumigatus* [10]
		Morphogenesis	*Candida albicans* [52]
Phosphatidylinositol family (PIPs)	Energy metabolism		*Candida albicans* [53]
	Affects fungal virulence	Morphogenesis	*Candida albicans* [54]
		CWI and MAPK signal transduction	*Candida albicans* [55]

## Data Availability

No new data were created or analyzed in this study.
